# Intrinsic and Extrinsic Methods for Augmenting Fracture Healing in the Hand and Wrist

**DOI:** 10.7759/cureus.74972

**Published:** 2024-12-02

**Authors:** Aryan Borole, Jack Trubiano, Marissa Viqueira, Karan Kalahasti, Thomas Stamos, David Kirschenbaum, Brian M Katt

**Affiliations:** 1 Department of Orthopedic Surgery, Rutgers Robert Wood Johnson Medical School, New Brunswick, USA

**Keywords:** accelerated fracture healing, bone morphogenetic protein, hand fracture, radiological and clinical fracture healing, wrist fracture

## Abstract

This review examines intrinsic and extrinsic augmentation techniques for uniting hand and upper extremity fractures, including bone morphogenic proteins (BMPs), platelet-rich plasma (PRP), low-intensity pulsed ultrasound (LIPUS), and pulsed electromagnetic fields (PEMF). While BMPs, PRP, LIPUS, and PEMF show potential in accelerating bone healing and reducing nonunion rates, their clinical adoption is limited by high costs and inconsistent results. This paper focuses on understanding the efficacy of these techniques, their drawbacks, and potential next steps for the field.

## Introduction and background

The first report of biophysical methods for fracture healing dates back to 1953 when Dr. Yasuda demonstrated that applying a direct current electrical field to fractured bones in rabbits could accelerate the healing process [1]. This marked the beginning of a new field of research into the use of biophysical methods, such as electromagnetic fields, ultrasound, intrinsic bone factors, and other non-invasive techniques, to enhance bone repair and regeneration.

Bone healing is a complex process involving cellular and physiological responses. In some cases, fracture union remains challenging and is ultimately delayed or not achieved, resulting in unfavorable economic or social outcomes. On the other hand, the scaphoid is of particular interest, as it accounts for the majority of all carpal bone fractures [2]. Recent studies have shown a 94% success rate in unstable scaphoid nonunions treated with screw fixation and grafting, highlighting the rare chance of persistent nonunion [3]. Often misdiagnosed as a wrist sprain, delayed treatment of scaphoid fractures increases the risk of nonunion and avascular necrosis in the proximal pole of the bone [3].

Another commonly fractured upper-extremity bone is the distal radius, which also carries a risk of nonunion. While incredibly rare, distal radius nonunion is clinically significant due to the high incidence of distal radius fractures. Accordingly, much of the previous work on fracture augmentation in the upper extremity focuses on the scaphoid and distal radius, though it also includes the phalanges.

This review article aims to describe the mechanisms and clinical applications of various methods of fracture augmentation, including biological enhancers, ultrasound, and electrical stimulation, to reduce the social and economic burden of hand and wrist fractures by promoting earlier healing and lowering nonunion rates.

## Review

Bone intrinsic factors

Bone Morphogenic Proteins

Bone morphogenic proteins (BMPs) are part of the transforming growth factor-beta (TGF-β) superfamily. They can accelerate healing and reduce the frequency of secondary interventions in cases such as acute tibial fractures [4]. There is growing interest in applying similar methods to enhance the healing of hand and wrist nonunions and improve fracture repair outcomes.

Most of the published research on BMPs focuses on treating nonunions in the hand rather than acute fractures. Two BMPs are clinically used for these nonunions: BMP-7 (also known as osteogenic protein-1 or OP-1) and BMP-2, which employs a collagen carrier to slow BMP release. BMPs recruit surrounding mesenchymal cells and induce their differentiation into osteoblasts, facilitating bone formation. Additionally, BMPs contribute to bone matrix production and vascularization.

We identified four studies investigating BMP-2 and two studies examining BMP-7 for hand and wrist nonunion healing, as detailed in Table 1 below.

**Table 1:** Published literature on the usage of BMPs for hand and wrist nonunion HO: heterotopic ossification, BMP: bone morphogenic protein, CT: computed tomography, ORIF: open reduction and internal fixation

Study	Morphogenic protein (dose)	Fracture	Sample size	How was the union assessed	Union rate	Complications from BMP usage
Bilic et al. 2006 [2]	BMP-7 (3.5 mg)	Scaphoid nonunion	10	X-ray	100%	0
Chevet-Noël et al. 2020 [3]	BMP-7	Scaphoid nonunion	5	X-ray	20%	0
Clark et al. 2022 [5]	BMP-2	Acute scaphoid fracture, scaphoid nonunion	7, 7	CT scan	100% , 86%	4 cases of HO
Ablove and Abrams 2015 [6]	BMP-2	Failed ORIF of scaphoid fracture	4	Radiograph and CT	100%	0
Brannan et al. 2016 [7]	BMP-2	Scaphoid nonunion	6	CT	17%	2 cases of persistent nonunion 4 cases of HO, 1 requiring revision surgery
Rice and Lubahn 2013 [8]	BMP-2 (4.2 mg)	Carpus (9), phalanx (7), distal radius (5), distal ulna (6) nonunion	27	Not reported	89% (24/27)	0

As BMP-7 has been only published in two studies on scaphoid nonunion with a total of 15 participants, it is difficult to draw concrete conclusions from the data. A study found that BMP-7 increases bone vascularization and supports nonunion healing, and the application of OP-1 with an allograft resulted in the healing of the scaphoid nonunion [2]. In this study, the authors defined nonunion as symptomatic nonunion of at least nine months duration with no evidence of progressive healing over the previous three months. Union was assessed on X-rays using plain film in four views by two independent, blinded radiologists who reviewed whether 0-33%, 34-66%, or 67-100% of the scaphoid bone surface that had been remodeled had healed. However, a more recent study suggested that there might be less benefit of BMP-7 usage for scaphoid nonunion, as only 1/5 of the study patients achieved union after a 10-year follow-up [3]. How nonunion was assessed in this study was not clearly defined, but a union was determined by reviewing wrist radiographs. Given the contrasting results, the evidence is unclear on the clinical benefit of BMP-7 usage for hand or wrist nonunion. Therefore, further research is needed to examine other treatment approaches, including new scaffolds combining BMP with other adjuncts.

Similarly, BMP-2 has been used to treat scaphoid nonunion following open reduction and internal fixation (ORIF). A recent study examined BMP-2 union rates in seven patients who were treated surgically prior [5]. From these, 6/7 cases with prior nonunion achieved union on average at 7.7 weeks. A smaller study yielded similar results, with 4/4 of patients with scaphoid nonunions achieving union in an average of 7.6 weeks [6]. While still small sample sizes, using BMP-2 can be a promising technique for managing a difficult clinical problem.

While BMPs can have benefits in healing following nonunion, it is also important to note the present challenges to their administration. These primarily include increased cost, limited licensing, and complications when used around joints and nerves. BMPs are FDA-approved for specific uses in long bones and spinal fusion surgeries. As a result, studies such as Brannan et al. cautioned against the off-label usage of BMPs and listed higher rates of complications than the previously stated studies [7]. The main complication involves heterotopic ossification, commonly noted in cases of BMP-2 [5,7]. In one case, the ossification was severe enough to warrant reoperation. Additionally, Rice and Lubahn found that radiographic union rates with BMP-2 were consistent with and not superior to previously published rates for nonunion repair of scaphoid, phalanx, and distal radius fractures [8].

The studies presented above all focused on nonunion healing. However, Clark et al. also looked at time to union for seven acute scaphoid fractures treated operatively [5]. They found that all seven acute fractures achieved union on average in 4.8 weeks. Given the small sample size, it is currently unclear whether this is a clinically relevant improvement in fracture healing, as scaphoid fractures currently have an 82% union rate six weeks after operative treatment [9]. A larger study by Rice and Lubahn looked at several upper extremity bones and found an 89% union rate overall, with an average union achieved 4.3 months after surgery [8].

Platelet-Rich Plasma

Regenerative medicine has introduced various innovative therapies to enhance fracture healing, including platelet-rich plasma (PRP) therapy. PRP therapy combines intrinsic growth factors such as platelet-derived growth factor, TGF-β, and insulin-like growth factor-1. Compared to other techniques, PRP has shown efficacy in reducing the risk of immune rejection and other complications associated with allogeneic or synthetic materials. PRP has gained attention for its potential to trigger the natural healing process by releasing biologically active factors and adhesion proteins, aiding in resolving chronic conditions [10].

Promising clinical data is supporting the potential of PRP to augment hand and wrist fracture healing. Namazi and Kayedi attempted to determine the intra-articular PRP injection's effect on acute, unilateral nondisplaced middle-third scaphoid fracture healing treated by casting [11]. While only seven patients in the experimental group were given a PRP injection and seven controls were given a saline injection, the healing rates were 7/7 and 6/7, respectively. CT scans were reviewed at two months, three months, and six months to monitor bone healing. At a six-month follow-up, the PRP group had statistically significantly better functional outcomes, with specific and usual activities scores higher in the PRP group and lower pain at rest, with no noted complications. However, there was no statistically significant difference in wrist motion, and while the cases healed faster than the controls, it was not statistically significant. Namazi and Mehbudi also published a similar study to evaluate the effect of PRP on functional results following intra-articular distal radius fractures [12]. Similar to their research on the scaphoid, they randomized 15 patients to receive intra-articular autologous PRP and 15 controls following closed reduction and percutaneous pinning in both groups. At three-month follow-up, the PRP group had less mean loss of flexion and wrist extension.

Zhong et al. also compared the clinical efficacy of open debridement screw fixation combined with bone grafting, percutaneous screw fixation, and percutaneous screw fixation combined with injection of PRP for the treatment of Slade and Dodds grades III to IV scaphoid nonunion [13]. A grade III nonunion has <1 mm sclerosis at the fracture site, while a grade IV nonunion is defined as having cystic formation present. The study included 25 and 28 grades III and IV fractures, respectively. The patients treated with percutaneous screw fixation and PRP injection reported significantly less pain and better wrist function seven days after surgery and significantly better visual analog scale and Mayo wrist scores at three months compared to the other two treatment methods. An interesting finding was that patients treated with open surgery debridement, bone grafting, and internal fixation who were radiologically found to have bone healing still reported wrist pain and limited joint activity. It was speculated that this was caused by joint capsule damage or scar contracture. This finding resulted in the authors concluding that percutaneous screw fixation with PRP injection could be a more effective method for treating grade IV scaphoid nonunion. While the efficacy of PRP injection in fractures other than grades III and IV will need further study, these results are promising for the improvement in functional outcomes post-injection.

Namazi and Kayedi, Namazi and Mehbudi, and Zhong et al. used an Arthrex double-syringe ACP® (autologous conditioned plasma) system for PRP preparation. At approximately $50 cost for the ACP® double syringe system, the cost of the syringe system is minimal. However, Peerbooms et al. reported that the cost for a single PRP injection is approximately $840.00 [14]. Given that it is an experimental treatment, it is unlikely that insurance companies will cover the treatment and have not covered it for other off-label uses.

Stem Cells

The first use of stem cells for fracture healing was reported in 2001 when a patient with an avulsed digital phalanx of the hand received stem cell therapy alongside traditional reconstructive surgical techniques [15]. After three months, the patient had successful functional outcomes, initiating further work in the field.

Mesenchymal stem cells (MSCs) are multipotent adult stem cells found in many tissues, including bone marrow and fat tissue, characterized by their ability to renew and differentiate into many cell types, including bone, cartilage, muscle, and connective tissue. While their efficacy has mostly been tested in the long bones, there is hope that their success can be replicated elsewhere, including the hand and upper extremities. In a trial in which 64 patients who had delayed healing of long bone fractures (humerus, radius, ulna, femur, tibia), 31 of the patients were randomized to receive MSCs differentiated in vitro to osteoblast precursors in an osteogenic differentiation medium before transplantation in the host [16]. After collecting and purifying the cells, they were injected into the fracture site using a radiation imaging instrument (C-arm). The injection of cultured osteoblast was based on the concept of bone marrow injection, which was supported by the theory that osteoprogenitor cells in bone marrow facilitate bone formation. Evaluation of the effectiveness of the cultured osteoblast injection was done using the modified callus formation score. No callus formation in one fracture cortex gave 0 points, slight callus formation gave 1 point, and bridging callus formation gave 2 points. The final, two-month average callus formation scores of the experimental and control groups were 7.1 and 5.8, respectively, which was statistically significant.

Extrinsic factors

Ultrasound Therapy

Ultrasound therapy, particularly low-intensity pulsed ultrasound (LIPUS), has emerged as a non-invasive modality with diverse medical applications, including enhancing bone healing, soft tissue regeneration, anti-inflammatory effects, and neuromodulation. LIPUS exerts its effects through several mechanisms: first, it enhances cellular activity at the fracture site, stimulating osteoblast proliferation and collagen synthesis, which are crucial for bone regeneration. Second, LIPUS reduces inflammation by modulating cytokine expression and promoting angiogenesis, facilitating faster tissue repair and reducing the risk of complications associated with delayed healing. Third, LIPUS enhances mineralized tissue formation, promoting early trabecular bridging and ensuring structural integrity during the healing process. These mechanisms collectively contribute to accelerated bone healing and improved clinical outcomes in fracture management [17].

Several key studies have investigated the efficacy of LIPUS in fracture healing, providing insights into its clinical benefits and potential applications. Kristiansen et al. conducted a prospective randomized study assessing LIPUS's impact on 61 patients with dorsally angulated distal radius fractures [18]. Treatment started within seven days post-fracture, with daily 20-minute sessions until a 10-week follow-up. The findings indicated a significant 38% reduction in healing time (61 ± 3.4 days compared with 98 ± 5.2 days) in the active ultrasound group compared to the placebo, attributable to accelerated trabecular healing, reduced loss of reduction, and enhanced trabecular bridging. These benefits were consistent across various patient and fracture characteristics, such as age, gender, volar angulation before reduction, Frykman score, involvement of the radio-ulnar and radiocarpal joints, displacement before the reduction, and fracture of the ulnar styloid process. These benefits suggested LIPUS's potential in modulating bone formation processes. Expanding on Kristiansen et al.'s work, Mayr et al. investigated LIPUS's efficacy in acute, stable scaphoid fractures [19]. This prospective randomized trial involved 30 patients divided into standard treatment and adjunctive ultrasound treatment groups. CT scans assessed healing biweekly and showed a faster healing rate in the LIPUS group (43.2 ± 10.9 days) compared to controls (62 ± 19.2 days, p<0.01). Trabecular bridging was significantly higher in the LIPUS group at six weeks post-injury (81.2% vs. 54.6%, p<0.05), reinforcing the earlier findings of accelerated bone healing with LIPUS.

Ricardo et al. extended LIPUS research to scaphoid nonunions treated with bone grafts [20]. In this double-blind trial with 21 male patients over an average follow-up of 2.3 years, those receiving LIPUS showed a significant reduction in healing time (56 ± 3.2 days vs. 94 ± 4.8 days, p<0.0001) compared to placebo. Both groups experienced improvements in pain levels and wrist motion, but the LIPUS group exhibited faster radiographic healing as assessed through X-ray. This suggests LIPUS's potential to reduce disability and healthcare costs associated with prolonged nonunion treatments, warranting further trials to optimize its role across different fracture types and surgical interventions.

Farkash et al. conducted a retrospective study on delayed union scaphoid fractures treated with LIPUS as an alternative to surgery [21]. Patients were categorized based on the time elapsed from injury to fracture diagnosis. The overall healing rate was 76%, with early-diagnosed patients (within three months) showing higher success (92%) compared to late-diagnosed patients (63%). Although not statistically significant (p=0.06), these results indicate LIPUS's efficacy, especially when treatment is initiated soon after injury, offering a potential non-invasive alternative to surgery.

Conversely, Basso and Pike evaluated LIPUS’s impact on wrist mobility following Colles fractures [22]. This study, involving 38 patients, focused on wrist range of motion as the primary outcome. Patients were randomly assigned to LIPUS or placebo groups, with a range of motion measured at plaster removal and two- and eight-week post-removal. Results showed no significant difference in median loss of motion percentages or physiotherapy referrals between the groups at any follow-up stage. This suggests that while LIPUS may accelerate bone healing, it might not significantly impact wrist motion recovery post-Colles fractures.

The reviewed studies collectively highlight LIPUS's potential in accelerating bone healing in specific upper extremity fractures, such as distal radial and scaphoid fractures. Furthermore, Palanisamy et al.'s overview underscored LIPUS's consistent efficacy across different fracture types, including lower and upper extremity fractures, supporting its potential as a standard adjunctive therapy in fracture management [17]. However, its efficacy in improving functional outcomes like wrist mobility remains inconclusive. Future research focusing on large-scale, multi-center trials to validate LIPUS's benefits across hand fracture types and investigating its mechanistic pathways is required. Additionally, exploring patient-specific factors influencing LIPUS efficacy will be crucial in optimizing treatment protocols.

However, the price of a LIPUS device can vary from $1300 to $5000, with little research being done to quantify the cost savings for hand fractures [23]. In 2019, the UK National Institute for Health and Care Excellence updated guidelines endorsing the EXOGEN® system for long bone nonunion fractures due to high healing rates and an estimated cost saving of £2,407 (~€2,648) per patient compared to current management, primarily by avoiding surgery. However, they found insufficient evidence to support its use for delayed healing fractures due to uncertainties about healing progression and potential surgery needs, resulting in varied cost outcomes [24]. LIPUS could potentially reduce the cost of hand treatments, but more research would be needed. In the United States, Medicare covers ultrasonic osteogenic stimulators under strict conditions, including documented nonunion and previous surgical intervention failure [25]. Many insurance plans cover EXOGEN®, though patient coverage can differ.

Pulsed Electromagnetic Fields

Pulsed electromagnetic fields (PEMF) are a non-invasive, extrinsic biophysical stimulation method believed to enhance osteogenesis and expedite the healing of bone and soft tissues. Since its FDA approval in 1979, PEMF has been extensively studied for its role in fracture healing, with a significant increase in high-quality research emerging in recent years. Clinically, PEMF has been employed for several decades to address orthopedic conditions such as bone formation, nonunions, and osteoarthritis. This technology can potentially optimize treatment through localized therapy delivery, thereby minimizing dose-dependent side effects. Despite its long-standing use, the efficacy, standardization, and underlying mechanisms of PEMF remain areas of ongoing investigation.

Peng et al. conducted a systematic review and meta-analysis of 22 randomized controlled trials involving 1,468 patients to assess the effects of PEMF on healing rates, healing times, pain, bone mineral density, and physical function across fractures, nonunions, and osteotomies [26]. The primary findings indicated that PEMF treatment significantly improved the overall fracture healing rate compared to control groups, with a risk ratio of 1.22 (95% CI: 1.10-1.35). Subgroup analyses demonstrated consistent benefits across various fracture ages, bone types, and injury methods. Delayed and unhealed fractures had a higher risk ratio of 1.64 (95% CI: 1.21-2.22) compared to fresh fractures (<6 weeks), which had a risk ratio of 1.20 (95% CI: 1.11-1.29), with similar heterogeneity in the data. Additionally, PEMF significantly reduced pain scores (standardized mean difference = -0.49; 95% CI: -0.88 to -0.10). It also showed a marginally significant effect on reducing healing time (standardized mean difference = -1.01; 95% CI: -2.01 to 0.00). While the evidence supporting PEMF’s ability to increase healing rates and reduce pain was of moderate quality, the evidence for accelerating healing times was very low quality. PEMF is a promising adjunctive treatment for enhancing bone healing and reducing associated pain. However, more high-quality trials are needed to evaluate its effectiveness and determine the optimal parameters, dose, and duration of PEMF therapy.

PEMF has shown promise in managing hand fractures. A prospective, double-blind, randomized, sham-controlled study by Factor et al. assessed the efficacy of adjunct PEMF therapy using the fracture healing patch (FHP) immediately after ORIF of acute distal radius fractures [27]. Patients with closed unilateral distal radial fractures meeting ORIF criteria received either a volar locking plate (VLP) with an active FHP or a VLP with a deactivated FHP (sham). The active FHP delivered continuous, localized PEMF therapy to the target fracture site 24 hours a day. The study aimed to accelerate fracture union and improve functional outcomes. Results showed significantly higher union rates at four weeks on CT scans in the active PEMF group compared to the sham group (70% vs. 54%, p=0.05). Four patients in the active FHP group achieved complete union (above 75% of bridging) at four weeks compared to none in the control group. Functional outcomes, including grip strength and patient-rated wrist evaluation function subscale scores, were also significantly better in the PEMF-treated group. No adverse events related to the FHP device were reported, suggesting PEMF therapy is a safe adjunct for enhancing distal radius fracture healing post-ORIF.

Additionally, PEMF has been studied in patients with delayed unions of phalanx fractures. De Francesco et al. conducted a retrospective study, administering PEMF to 43 patients in eight-hour intervals daily for 60 days. The study reported a 25% higher rate of complete bone union within 12 weeks compared to the control group (n=37) and found PEMF to be most effective in diaphyseal fractures, promoting earlier mobilization and improved range of motion (p=0.0534) [28].

Insurance coverage for PEMF and associated electrical stimulation devices varies. The Centers for Medicare and Medicaid Services (CMS) cover device usage only under limited circumstances of fracture nonunion. CMS defines nonunion as existing when serial radiographs confirm that fracture healing has ceased for three or more months prior to starting treatment with an electrical osteogenic stimulator. Serial radiographs must include at least two sets, each with multiple views of the fracture site, separated by at least 90 days. Private insurers have similar parameters, typically covering PEMF only for delayed unions, failed arthrodesis at high-risk sites (e.g., open or segmental tibial fractures, carpal navicular fractures, fifth metatarsal fractures, distal radius, and sesamoid bones), or congenital pseudarthrosis with no X-ray evidence of healing progression for three or more months despite appropriate care. Even if deemed medically necessary, insurance providers usually do not fully cover PEMF devices. Medicare patients typically pay 20% of the allowable cost for bone growth therapy devices, while costs for other insured patients vary by provider.

While promising, the variability in study designs, PEMF parameters (frequency, intensity, duration), and outcome measures across studies warrants caution in interpreting the data. Despite some studies indicating moderate to high-quality evidence for certain outcomes, the overall evidence base is heterogeneous, underscoring the need for larger, well-designed trials. Clinical application of these devices is also limited by the narrow circumstances within which insurers will cover such a device. In conclusion, the literature reviewed suggests that PEMF holds promise in accelerating bone healing, reducing pain, and improving functional outcomes following fractures. However, standardization of PEMF protocols and further rigorous research, including pre-clinical studies, are essential to validate these findings and optimize treatment strategies.

Challenges and opportunities

Although shorter fracture-healing times provide significant health and socioeconomic benefits, gaps remain in research on the effectiveness of these interventions and their adoption by clinicians. For instance, despite positive outcomes reported in studies, clinical adoption of PEMF for fracture augmentation remains limited. Bhavsar et al. identified 141 relevant publications, with animal studies predominantly using direct current stimulation and clinical studies favoring PEMF [29]. The review also included a survey of 161 orthopedic surgeons worldwide, revealing that although most were aware of the benefits of electrical stimulation, only one-third had utilized it. High costs and inconsistent results were cited as primary barriers. Despite these challenges, 85% of surgeons indicated they would consider using electrical stimulation if a cost-effective, easy-to-use device became available.

Higher complication rates and unclear clinical evidence further discourage physicians from adopting some of these techniques. For example, BMPs, the most extensively studied biological agents for enhancing bone repair, are not widely used. In addition to potential side effects, BMPs have other drawbacks that limit their widespread application. They are expensive due to the high doses required for treatment, which often exceed the physiological concentrations of these proteins. Although studies are investigating BMP use in upper extremity fractures, the FDA currently approves BMPs only for treating long bone nonunion, acute tibial fractures managed with intramedullary fixation, and spinal fusion surgeries.

There may be additional benefits to combining fracture augmentation techniques, particularly when they utilize different mechanisms of action. An animal study on rats with femoral osteotomies stabilized using external fixators demonstrated the beneficial effects of localized MSC injections combined with PTH therapy [30]. The combination treatment led to increased callus formation compared to controls and produced a dose-dependent response to PTH. Results showed that combined therapy accelerated fracture healing and enhanced bone properties more effectively than single-agent therapy. While these findings remain limited to animal models, investigating combination therapies in human prospective studies would be a valuable next step.

## Conclusions

This review examines techniques for augmenting fracture healing in the hand and upper extremities, focusing on BMPs, PRP, LIPUS, and PEMF. While these methods show promise in reducing nonunion rates and accelerating healing, their widespread adoption is hindered by high costs, inconsistent results, and limited clinical use. BMPs and PRP demonstrate potential for specific fracture types but face challenges, including complications and uncertain benefits in broader applications. LIPUS and PEMF show efficacy in expediting healing and reducing pain, but variability in treatment protocols limits their broader implementation. This review highlights the need for large-scale studies and suggests exploring combination therapies to optimize clinical outcomes.
